# Role of N Doping in the Reduction of Titania Nanostructures

**DOI:** 10.1021/acs.jpcc.3c04665

**Published:** 2023-10-02

**Authors:** Elena R Remesal, Ángel Morales-García, Francesc Illas

**Affiliations:** Departament de Ciència de Materials i Química Física & Institut de Química Teòrica i Computacional (IQTCUB), Universitat de Barcelona, c/Martí i Franquès 1-11, 08028 Barcelona, Spain

## Abstract

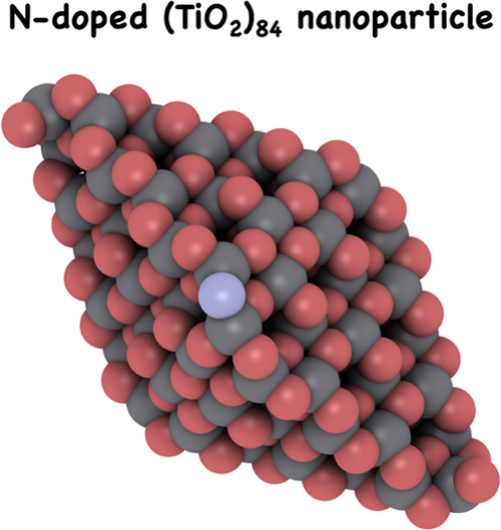

The effect of N-doping of titania (TiO_2_) nanoparticles (NPs) on their reduction through neutral O vacancy (O_vac_) formation is investigated using all electron density functional theory-based calculations, including hybrid density functionals, and taking the bipyramidal anatase (TiO_2_)_84_ NP as a realistic model. The location of the N dopant is systematically analyzed, including O substitution in the (TiO_2_)_84_ structure and N occupying interstitial regions. Our computational study concludes that interstitial N doping is more favorable than N substituting O atoms and confirms that the presence of N reduces the energy gap. In the N-doped NP, O_vac_ formation is more favored than in undoped NP but less than in the N-doped bulk, which has important consequences.

## Introduction

During the last decades, an intense research activity has been carried out around semiconducting transition metal oxides (i.e., TiO_2_, ZnO, WO_3_) and their possible heterostructures formed by mixing configurations.^1^ These materials constitute the leading families of photocatalytic materials with implications in energy and environmental science.^2,3^ Since the pioneering works by Fujishima and Honda^4^ and Schrauzer and Guth,^5^ TiO_2_ has become one of the most promising photoactive materials, especially when nanostructured, due to its intrinsic electronic properties attributed to a plentiful availability, nontoxicity, chemical stability, and high reactivity.^6^ Nevertheless, TiO_2_ exhibits important limitations such as a wide energy gap and fast recombination of photogenerated electrons and holes, which cause a restrictive light absorption.^7,8^ These drawbacks have greatly motivated further research aimed at developing and designing TiO_2_-derived materials active in the visible-light region of the electromagnetic spectrum with low recombination.^9−11^

In the last years, numerous theoretical and experimental studies on TiO_2_ have been published following different strategies to overcome these drawbacks, aiming at improving the photocatalytic performance.^12−16^ One of these strategies is doping stoichiometric TiO_2_ polymorphs with nonmetals such as C, N, or S.^17−20^ Nonmetal doping in the main has been found to be more favorable than doping with the metal counterpart due to the low rate of recombination center formation, where the photogenerated species are annihilated. Furthermore, electronic analysis confirms the presence of additional electronic states in between the valence and conduction bands of doped TiO_2_ promoting the reduction of the energy gap and, consequently, the activation under visible-light illumination.^10^

Most of these investigations focused on the structural and electronic properties, and just a few of them paid attention to how nonmetal doping affects the TiO_2_ reducibility through the formation of oxygen vacancies (O_vac_). In this context, Di Valentin et al.^21^ have investigated the effect of carbon impurities on the electronic band structure of bulk titania polymorphs (i.e., anatase and rutile) with respect to the oxygen partial pressure. Those results reported that the oxygen vacancy formation energy decreases up to 20% in the presence of interstitial carbon and more than 50% in the presence of substitutional carbon. This effect might be ascribed to the tendency of substitutional C atoms to accept the excess electrons generated from oxygen vacancy formation. These authors also investigated the electronic and optical properties of bulk TiO_2_ modified by substitutional N.^22^ From first-principles calculations, they concluded that N-doping leads to different behaviors depending on the crystalline structure. For instance, N-doping promotes red and blue shifts of the absorption edge in anatase and rutile, respectively, thus inducing different levels of photoactivity. A subsequent study aimed at characterizing the nature of paramagnetic species in N-doped TiO_2_ polymorphs^23^ concluded that one of the consequences of nitrogen doping is the reduction in the formation energy of an oxygen vacancy, in some cases by up to 80%. The energetically favorable charge transfer from Ti^3+^ ions to N results in a lower formation of the oxygen vacancy compared to the undoped counterpart.^24^ This finding is puzzling because, as oxygen vacancy sites are efficient electron traps, it seems to indicate that N-doped titania, even exhibiting a reduced gap, will easily form electron traps that are detrimental to the photocatalytic activity.

Several additional publications have characterized the effect of the dopant location on TiO_2_ surfaces and nanostructures. Recently, Di Liberto et al.^25^ investigated the N-doping of the exposed (001)/(101) surfaces of anatase TiO_2_ heterostructures. They found that N at the (001) side of the interface constitutes the most stable doping arrangement. On the other hand, Kakil et al.^26^ demonstrated that subsurface N doping in TiO_2_ anatase is depth-dependent for both substitutional and interstitial N, and that N location depends on the nanocrystal facet. Moreover, they investigated N-doping of Ti_9_O_18_ and Ti_28_O_56_ nanoclusters and found that the interstitial N formation energy is lower than that of substitutional N. Additional theoretical calculations predicted a decreasing of the band gap due to the presence of nitrogen, in agreement with results from UV–vis spectroscopy.^27^ Combining the density functional theory (DFT) calculations and extended X-ray absorption fine structure (EXAFS) techniques, Ceotto et al.^28^ studied the nitrogen location in nanocrystalline N-doped TiO_2_ and concluded that, at a low concentration of N-doping, N atoms substitute O atoms, whereas at higher N-doping concentrations, oxygen vacancies appear.

A common feature of most of the previous computational studies is the use of bulk or extended surface models. However, in real nanostructured finite systems, exhibiting a variety of morphologies,^29^ extended surface models represent solely their facet regions, but cannot provide information regarding other regions such as edges, corners, or apical sites, which can actively participate in the formation of the N-doped titania nanostructures as well as in the vacancy formation thereon.^30^ Hence, the use of realistic nanostructure models appears necessary to account for the chemistry of these systems. The goal of the present study is precisely to analyze N-doping in TiO_2_ NPs and its effect on the oxygen vacancy formation.

## Computational Strategy and Titania Models

**Figure 1:**
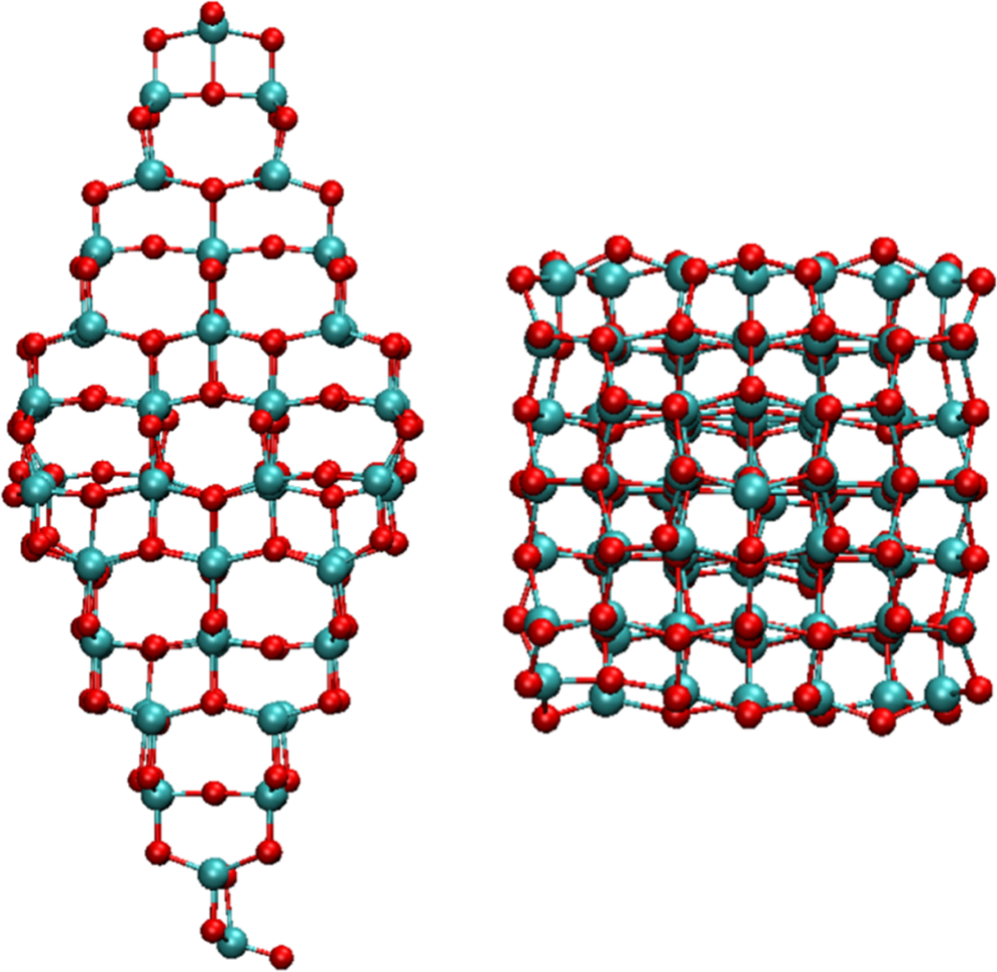
Side and top views of (TiO_2_)_84_ NP. This nanostructure exhibits a bipyramidal morphology with (101) surfaces in the eight facets. Red and cyan spheres correspond to oxygen and titanium atoms.

**Figure 2:**
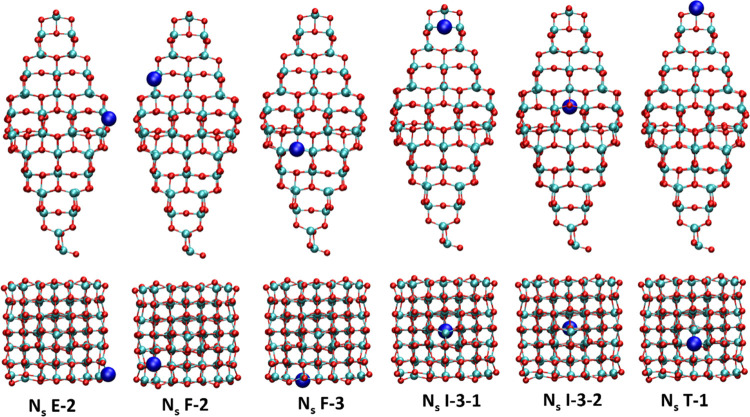
Side and top views of the representative optimized *N*_s_-doped (TiO_2_)_84_ NPs. Cyan, red, and blue spheres denote titanium, oxygen, and nitrogen atoms, respectively. Note that the N atom has been represented by a double radius to better visualize the doping site.

**Figure 3:**
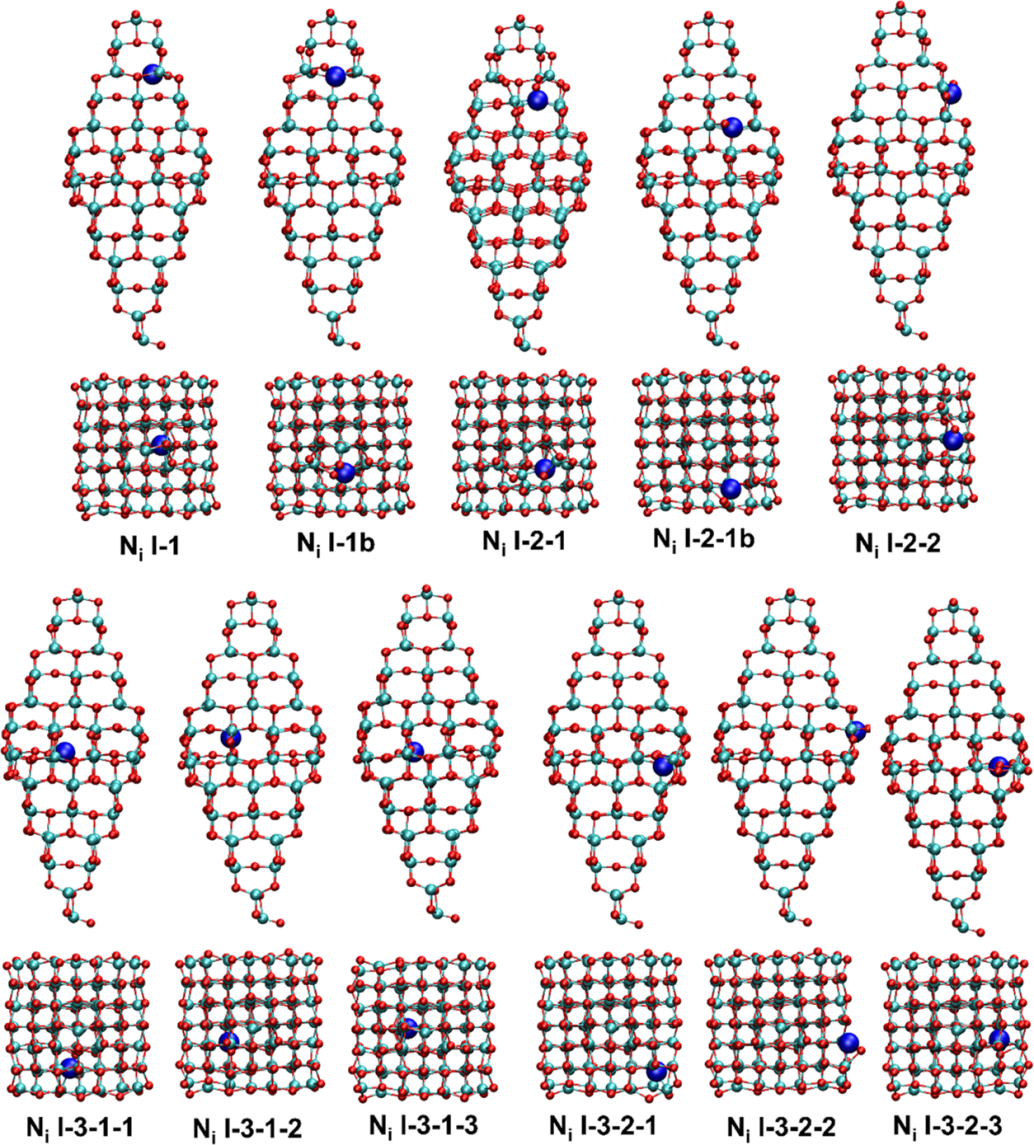
Side and top views of the representative optimized *N*_i_-doped (TiO_2_)_84_ NPs. Note that N atoms have been represented with a double radius to better visualize the doping site. The color scheme is similar to that in Figure 2.

The realistic bipyramidal (TiO_2_)_84_ anatase nanoparticle (NP), well-defined in the scalable regime^30^ with a Wulff-like equilibrium geometry^31^ (Figure 1), has been selected to carry out a systematic exploration on N-doping and its effect on its reducibility. This NP model is representative of those found in most photocatalytic oriented experiments^29,32^ that do not rely on the use of single-crystal samples with well-defined surfaces. This (TiO_2_)_84_ bipyramidal model exposes the most stable (101) facets, only conferring it a high structural stability. On this model, different regions are selected to generate the N-doped nanostructures. The investigated regions are located in apical (labeled as T), edge (E), facet (F), and inside (I) portions, as shown in Figure 2. Here, one needs to distinguish different O atoms as there are different atomic environments. In particular, O atoms with 2-fold (O_2c_) and 3-fold (O_3c_) coordination; the former are located at the shell of the bipyramid (i.e., apical, edges, and facets), while the latter are mainly located at the core of the nanoparticle. On the other hand, the interstitial dopant is located inside the channel of the structure visualized in the side view (Figures 1 and 3). Further details regarding the notations used to describe the relevant atomic environment are introduced later and clearly explained for each particular case. To inspect the stability and electronic structure of the N-doped (TiO_2_)_84_ NP, we perform density functional theory (DFT)-based calculations by explicitly including all electrons with the electron density described through a numerical atom-centered orbital (NAO) basis set as implemented in the Fritz Haber Institute *ab initio* molecular simulations (FHI-aims) code.^33^ The Perdew–Burke–Ernzerhof (PBE)^34^ density functional within the generalized gradient approximation (GGA) was selected to describe the thermodynamic stability of N-doped titania nanostructures. Calculations were carried out using the tier-1/light grid basis set. For TiO_2_ systems, this setting leads to results of Gaussian type orbital (GTO) valence triple-ζ plus polarization basis set quality.^35^ The convergence threshold for atomic forces in relaxation of all atoms in the N-doped and pristine (TiO_2_)_84_ nanostructures was set to 10^–3^ eV Å^–1^. Due to the presence of relatively heavy Ti atoms, relativistic effects were explicitly included through the zero-order regular approach (ZORA).^36,37^

To examine the stability of N-doped TiO_2_ NPs, we consider the formation energy of substitutional (*N*_s_) and interstitial (*N*_i_). The formation energy labeled as *E*_f_ is defined as
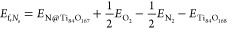
1
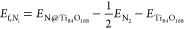
2where *E*_N@Ti_84_O_167__ and *E*_N@Ti_84_O_168__ correspond to the total energies of substitutional and interstitial doped NPs, respectively; *E*_Ti_84_O_168__ is the total energy of the undoped NP; and *E*_N_2__ and *E*_O_2__ are the total energies of the N_2_ and O_2_ molecules in their electronic ground state, which for the O_2_ molecule is a triplet state. This definition of reference state of element is consistent with previous studies.^19,26,35^ Consequently, the nitrogen molecule is taken as a convenient reference, and from the energy balance in eqs 1 and 2, we do not expect any difference when employing a different source of nitrogen. In fact, different nitrogen precursors are used in experimental studies such as ammonium hydroxide or ammonia. However, these lead to additional products, thus complicating the underlying basic chemistry, and, therefore, are not considered in the present work.

On the other hand, the oxygen vacancy formation energy *E*_f,v_O__ in doped titania nanostructures is defined as
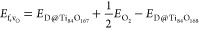
3where *E*_D@Ti_84_O_168__ and *E*_D@Ti_84_O_167__ are the total energies of a doped (TiO_2_)_84_ NP, with either substitutional or interstitial N, and its reduced counterpart, respectively. In all cases, the titania nanostructures are fully relaxed. According to eq 3, *E*_f,v_O__ > 0 corresponds to the extra energy required for removing a neutral single oxygen relative to the O_2_ molecule; in other words, the energy required to reduce the N-doped (TiO_2_)_84_ NP.^38^

To analyze the electronic structure, it is necessary to recall that generalized density functionals such as PBE systematically underestimate the energy gap of semiconducting materials.^39^ To overcome this deficiency, we rely on the PBEx hybrid density functional containing 12.5% of Fock exchange, which accurately reproduces the structural and electronic properties on anatase and rutile bulk titania phases.^40^ Since the PBE structures for these systems are generally accurate,^41^ single-point calculations with the PBEx functional are carried out at the PBE geometry. Analysis of the effect of the N atom on the energy gap relies on the Kohn–Sham energy levels of the frontier orbitals instead of density of states (DOS) plots. This is because the scrutinized systems are finite and, hence, exhibit discrete levels, and because a DOS analysis includes an artificial broadening that can somehow bias the results.

## Results and Discussion

### Formation of N-Doped (TiO_2_)_84_ Nanostructures

The formation of N-doped titania nanostructures is investigated using the (TiO_2_)_84_ NP depicted in Figure 1 as a suitable model and analyzing O substitution by N (*N*_s_) and interstitial N (*N*_i_). In each case, various configurations exist, which are analyzed systematically following that recently published by some of us investigating C-doped TiO_2_ NPs.^19^ For N_s_, six different positions in the apical (T), facet (F), edges (E), and inside (I) of the NP are investigated as highlighted in Figure 2. The formation energy of the corresponding N-doped NPs is reported in Table 1, where values are in the 4.70–5.44 eV range. Thermodynamically speaking, the I-3-1 N-doped configuration is the one most favorable, while T-1 is the less one. These results are consistent with a previous analysis of C-doped (TiO_2_)_84_ NPs,^19^ where the I-3-1 C-doped NP is the most stable regardless of the oxygen chemical potential.

**Table 1:** Formation Energy of N-Doped (TiO_2_)_84_ NPs Calculated Following Equations 1 and 2 a

doping site	*E*_f_ (eV)	doping site	*E*_f_ (eV)
N_s_-E-2	5.05	N_i_-I-1	4.09
N_s_-F-2	5.16	N_i_-I-1b	2.35
N_s_-F-3	4.90	N_i_-I-2-1	3.20
N_s_-I-3-1	4.70	N_i_-I-2-2	2.62
N_s_-I-3-2	5.06	N_i_-I-2-1b	2.96
N_s_-T-1	5.44	N_i_-I-3-1-1	5.36
		N_i_-I-3-1-2	3.59
		N_i_-I-3-1-3	4.20
		N_i_-I-3-2-1	4.15
		N_i_-I-3-2-2	2.76
		N_i_-I-3-2-3	4.08

a *N*_s_ and *N*_i_ denote substituting O by N atom and interstitial positions, respectively.

At this point, it is interesting to compare the cases of N- and C-doping. The formation of *N*_s_-doped titania nanostructures is energetically more favorable than the formation of C-doped ones (*C*_O_).^19^ Although there is no clear correlation between the formation energies of N- and C-doped (TiO_2_)_84_ NPs, the N-doped titania formation energy range is almost twice lower than that corresponding to C-doped NPs. Moreover, it is also of interest to compare our results with those involving N-doping of bulk and extended surface models. For the anatase phase, the reported *N*_s_ formation energy is ∼5 eV, independently of nitrogen concentration.^42^ For the (001) and (101) anatase surfaces, the most stable position is located near the interface region, although the location on the (101) surface is thermodynamically preferred than on the (001) one.^25^ In general, the present results for finite titania NPs show similar *N*_s_ formation energies to those in bulk and surface models, with values of ∼5 eV. This seems to indicate that the (TiO_2_)_84_ NP is large enough to mimic the facet sites of extended surfaces.

We now focus on N-doping at interstitial regions (*N*_i_) as depicted in Figure 3. Here, the N atom localizes in one of the existing four channels (see Figure 1, side view) moving from the apical to the equatorial region. The number of sites able to accommodate the N atoms increases in the same direction, as expected from the increasing thickness at the equatorial region where the N atom finds a larger space. This is the reason why there is only one site in the apical region labeled as *N*_i_-I-1, and three sites in the equatorial channel (*N*_i_-I-3-2-3). All of these sites are located inside the nanoparticle and thus, only the I capital letter is used to denote them, with the three digits indicating the position with respect to the apical region in terms of proximity, the interstitial channel, and the position inside the channel, respectively. In some cases, the b letter is included in the notation to indicate that the N is near the surface of the (TiO_2_)_84_ NP. Table 1 lists the formation energies of N-doped titania nanostructures in the interstitial region ranking between 2.35 and 5.36 eV. The structures I-1b, I-2-2, and I-3-2-2 are the most favorable situations showing the lowest formation energies of 2.35, 2.62, and 2.76 eV, respectively. From these results, one may conclude that the *N*_i_ is energetically more favorable than *N*_s_. Compared to a similar case of C-doping (*C*_i_), it is clear that *N*_i_ is by far more favorable and the *C*_i_ formation energies are around 8.5 eV.^19^ The comparison to the results from extended models is also interesting. There, *N*_i_ doping of the anatase bulk phase requires 4.3 eV, whereas a lower value of 3.7 eV is required to insert *N*_i_ at surface sites. Clearly, the formation of *N*_i_-doped titania nanostructures is more favorable than either the bulk or the extended surface site, although for such extended systems *N*_i_ is also preferred to *N*_s_.^42^

**Figure 4:**
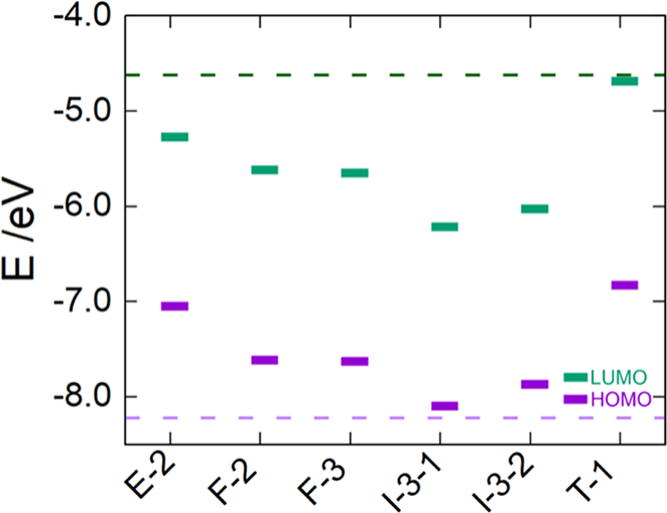
Kohn–Sham orbital energy level diagram of *N*_s_-doped (TiO_2_)_84_ NPs obtained from DFT based calculations through the hybrid PBEx functional. The dotted lines correspond to Kohn–Sham orbital energy levels of bare (TiO_2_)_84_ NP.

**Figure 5:**
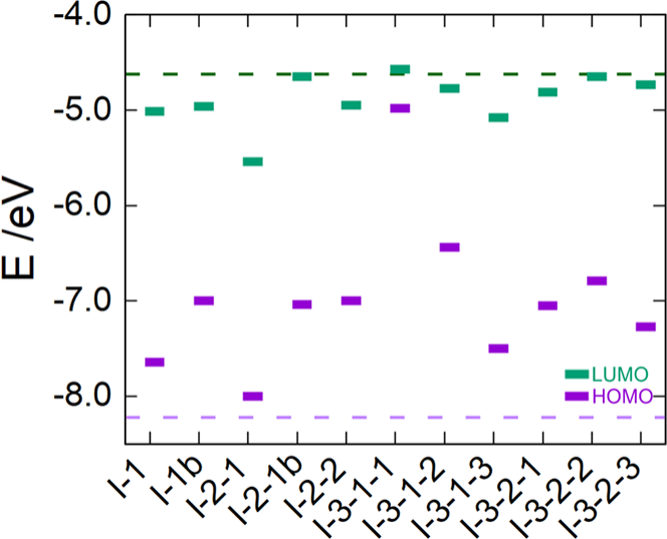
Kohn–Sham orbital energy level diagram of *N*_i_-doped (TiO_2_)_84_ NPs obtained from DFT based calculations through the hybrid PBEx functional. The dotted lines correspond to Kohn–Sham orbital energy levels of bare (TiO_2_)_84_ NP.

Once we have discussed the formation of N-doped (TiO_2_)_84_ NPs, it is important to analyze the effect that N doping has on the resulting electronic structure. To this end, we focus on the electronic structure arising from the hybrid PBEx density functional. As expected, the presence of N in the (TiO_2_)_84_ NP results in addition of energy levels with concomitant electronic modifications that decrease the energy gap with respect to the undoped (TiO_2_)_84_ NPs (see Table S1 of Supporting Information, SI). This effect has also been reported for the C-doped (TiO_2_)_84_ NP, but with significant differences. In general, C doping leads to a larger reduction of the energy gap. In the N-doped structures, *N*_s_ promotes a stabilization of the Kohn–Sham LUMO orbital energy relative to that of the undoped NP, resulting in the reduction of the energy gap at 1.9 eV, almost half of the value corresponding to the undoped nanostructure (3.60 eV). This trend is systematically observed except for the T-1 site, as shown in Figure 4. On the other hand, in most of the *N*_i_-doped (TiO_2_)_84_ NPs, the energy gap decreases due to the addition of extra electrons, which raises the HOMO levels (Figure 5). The lowest energy gap is found for I-3-1-1 and I-3-1-2 in the equatorial cases (0.41 and 1.68 eV), whereas it is larger for the edge E-2 (1.78 eV) and inside I-3-1 and I-3-2 regions (1.88 and 1.83 eV).

Here, one may wonder whether the effect of the N doping is analogous (or not) to the effect of another nonmetal doping such as C. To answer this question, we extend our previous analysis^19^ to investigate how doping affects the electronic structure and to make systematically the comparison between these two cases. The calculations of C-doped titania nanostructures were carried out without using spin-polarized calculations because C has an even number of electrons different from N that exposes an unpaired number as mentioned earlier. This extended analysis includes the formation of titania-doped structures by substitutional C atoms replacing O atoms, *C*_O_, and interstitial C atoms, *C*_i_, both having similar effects (see Figures S1 and S2). For instance, consider *C*_O_ for the I-3-2 inside, T-1 apical, and F-3 facet together with the I-1, I-2-1, I-2-2, I-3-1-1, I-3-1-3, I-3-2-1, and I-3-2-2 interstitial sites. This notation follows the same sequence described earlier when discussing N-doping at interstitial regions. In general, the LUMO energy orbital is unaltered by the presence of the C doping, but in most of the cases, the HOMO energy rises due to localized occupied states in the energy gap. These impurity states modify the absorption range, which is consistent with the experimental absorption edge shift toward the visible up to almost 1.7 eV.^43^ In general, the energy gap decreases more than 2 eV with respect to (TiO_2_)_84_ NP by the presence of the C-doping agent. The face and apical position of *C*_O_, F-2 and T-1, have the lowest energy gap of 0.88 and 0.83 eV, respectively, followed by the internal position, I-3-2, with 1.13 eV. This is the only case where the LUMO energy orbital is stabilized. Finally, it is necessary to point out that the largest reduction of the energy gap is observed when the C atom locates in the interstitial regions close to the equatorial area, like I-3-1-1, I-3-1-2, I-3-1-3, and I-3-2-2, whose formation energy ranges between 0.22 and 0.31 eV.

### Formation of Reduced N-Doped (TiO_2_)_84_ Nanostructures: Formation of Oxygen Vacancies

It has been well established that the presence of certain dopants on semiconducting oxide materials favors somehow their reducibility by facilitating the generation of oxygen vacancies.^22−24^ It has previously been shown that the formation of oxygen vacancies (O_vac_) in undoped (TiO_2_)_84_ NP requires energies between 3.63 and 4.54 eV.^38^ In that study, the NP region where vacancy creation is the most favorable is in the subsurface region. Taking advantage of our previous analysis on the bare system, we investigate here the influence of N-doping on the reducibility of the N-doped (TiO_2_)_84_ NP. For completeness, we also comment on the influence of C-doping on the NP reducibility.

**Figure 6:**
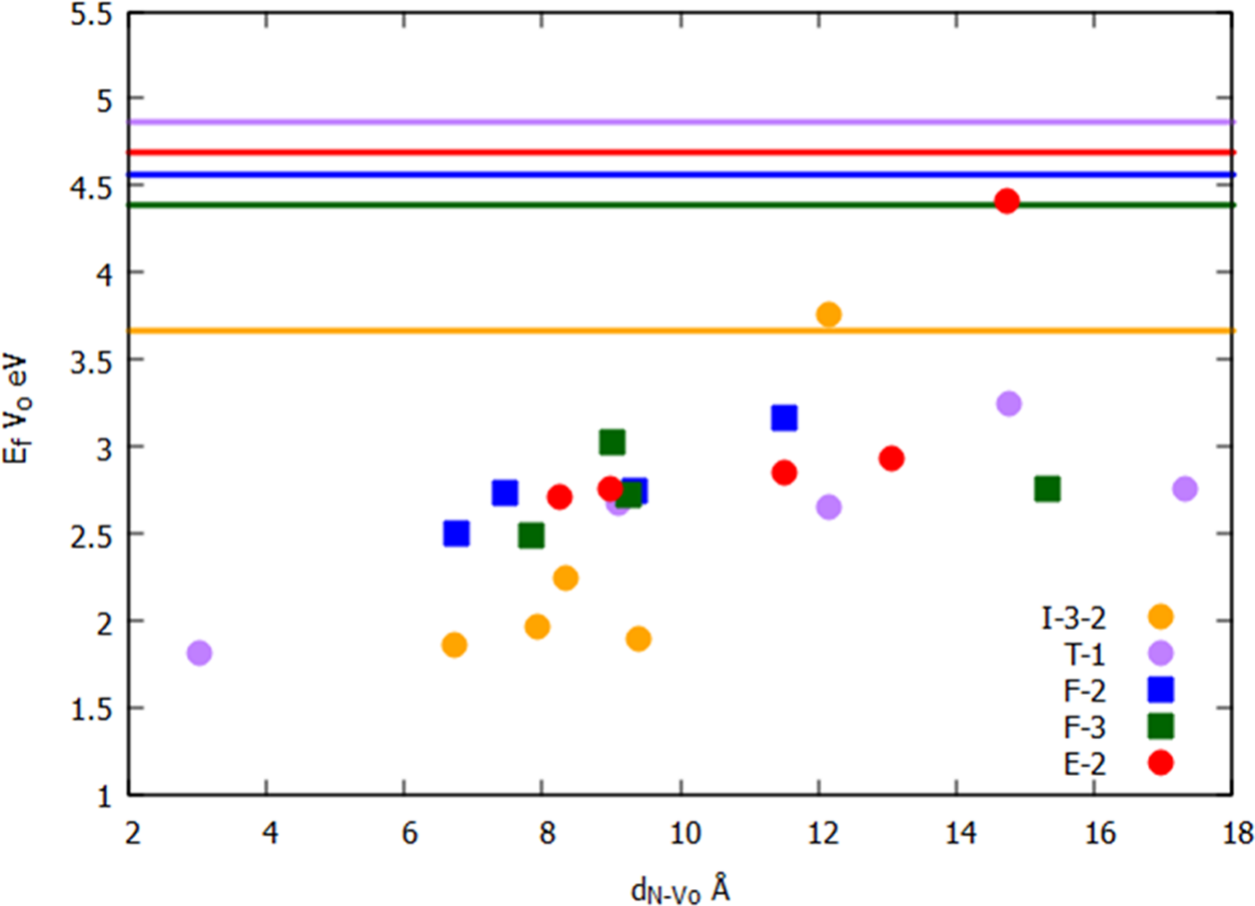
Trends between *E*_f,v_O__ and *d*_N-Vo_. The solid lines correspond to *E*_f,v_O__ in bare (TiO_2_)_84_ NP. (See ref (38).).

Studying the effect of *N*_s_ doping on O_vac_ formation required fixing the N atom in one of the following sites: T-1, E-2, F-2, F-3, I-3-1, and I-3-2; then, the octahedral O atom was removed from the other sites. Therefore, for each N-doped (TiO_2_)_84_ NP, one has five different sites to create O_vac_. For example, one can locate the N atom in the E-2 site and the O_vac_ can be generated in T-1, F-2, F-3, I-3-1, or I-3-2 site. An analogous strategy is followed in case of *N*_i_ doping here, fixing the N position and considering O_vac_ formation on the possible symmetry distinct sites. The results of the corresponding series of calculations are listed in Tables 2 and 3. Starting with the *N*_s_ case (Table 2), the O_vac_ formation energy ranges between 1.81 and 5.15 eV, with most cases exhibiting formation energies below 3.80 eV, thus confirming that the presence of N in the structural framework of the (TiO_2_)_84_ NP favors its reducibility, as found for periodic models.^22−24^ More importantly, by comparing the O_vac_ formation energies of the titania nanostructures,^38^ it turns out that this reduction is larger in the case of the TiO_2_ NPs. To rationalize somehow this low O_vac_ energy formation in the N-doped NP, we consider the distance between the vacancy and the N dopant atom, denoted as *d*_N-Vo_, which is also listed in Table 2. Unfortunately, there is no systematic correlation of all cases investigated, but interesting information emerges when analyzing each case individually. There are six cases in which the lowest *E*_f,v_O__ correlates with the shortest *d*_N-Vo_ distance. Using a O_vac_/N-atom notation to define simultaneously the position of O_vac_ and N atom, the six cases above are E-2/I-3-2, F-2/I-3-2, F-3/I-3-2, E-2/I-3-2, I-3-2/F-2, and T-1/I-3-1. In this shortlist, the *E*_f,v_O__ is between 1.81 and 2.71 eV. Interestingly, the inside regions are consistently involved in all of these cases. The I-3-2 site is able to hold the doping of N atom by substituting the O atom. If we spotlight on the oxygen vacancy energy formation on face (F-2, F-3) and apical (T-1) regions, we can observe a correlation between the vacancy energy formation and *d*_N-Vo_ distance, which grows consistently as *d*_N-Vo_ grows (see Figure 6). Compared to the undoped nanostructures, O_vac_ formation (solid lines in Figure 6) is energetically more favorable in N-doped titania nanostructures by ∼1 eV. Finally, for the *N*_i_-doped (TiO_2_)_84_ NPs, Table 3 indicates a vacancy formation energy between 2.11 and 4.91 eV. As mentioned earlier, the presence of *N*_s_ favors the NP reducibility, but its presence in the interstitial region has a less effect. The most favorable situation gives formation energies of 2.11 and 2.86 eV, when the vacancy locates at the internal position (I-3-2) or apical (T-1) area. Also, the I-3-1 vacancy position is the only one that is practically unaffected by the dopant agent. Finally compared with previous studies, for the *N*_i_ cases, the presence of the dopant has less effect on reducibility than reported for the bulk, where the oxygen vacancy formation energy in the presence of *N*_i_ was around 0.6 eV.^24^

**Table 2:** Formation Energy of the Oxygen Vacancy (*E*_f,v_O__, in eV) of *N*_s_-Doped (TiO_2_)_84_ NP^a^

O_vac_	N site	d_N-Vo_	*E*_f,v_O__	O_vac_	N site	d_N-Vo_	*E*_f,v_O__
E-2	F-2	11.50	2.85	I-3-1	E-2	13.16	4.33
	F-3	9.00	2.76		F-2	7.59	3.76
	I-3-1	13.06	2.93		F-3	15.42	3.84
	I-3-2	8.26	2.71		I-3-2	9.46	3.73
					T-1	3.16	5.15
F-2	E-2	11.50	3.16	I-3-2	E-2	8.33	2.24
	F-3	9.33	2.75		F-2	6.73	1.86
	I-3-1	7.47	2.73		F-3	7.92	1.96
	I-3-2	6.75	2.50		I-3-1	9.39	1.90
					T-1	12.15	3.76
F-3	E-2	9.03	3.03	T-1	E-2	14.75	3.25
	F-2	9.24	2.72		F-2	9.12	2.68
	I-3-1	15.32	2.76		F-3	17.30	2.76
	I-3-2	7.84	2.49		I-3-1	3.03	1.81
					I-3-2	12.16	2.65

a The O_vac_ and N notation indicates the sites in the nanoparticle where the oxygen vacancy and the N doping locates, respectively. The *d*_N-Vo_ (in Å) corresponds to the distance between O_vac_ and N atom.

**Table 3:** Formation Energy of the Oxygen Vacancy (*E*_f,*v*_O__, _i_n eV) of *N*_i_-doped (TiO_2_)_84_ NP^a^

O_vac_	N site	d_N-Vo_	*E*_f,*v*_O__	O_vac_	N site	d_N-Vo_	*E*_f,*v*_O__
E-2	I-1	10.60	3.33	I-3-2	I-1	6.78	2.25
	I-1b	9.95	3.82		I-1b	6.99	2.86
	I-2-1	8.03	3.22		I-2-1	5.74	2.40
	I-2-1b	5.22	3.66		I-2-1b	5.17	2.85
	I-2-2	4.59	3.23		I-2-2	5.97	2.11
	I-3-1-2	8.94	4.12		I-3-1-2	2.74	3.33
	I-3-2-2	3.14	4.08		I-3-2-2	6.41	2.97
F-2	I-1	6.94	3.19	I-3-1	I-1	3.15	3.63
	I-1b	4.95	3.63		I-1b	3.70	4.91
	I-2-1	5.96	3.10		I-2-1	5.10	4.30
	I-2-1b	6.26	3.61		I-2-1b	8.67	4.73
	I-2-2	10.90	3.13		I-2-2	6.98	4.09
	I-3-1-2	5.63	3.36		I-3-1-2	10.45	4.78
	I-3-2-2	11.99	3.73		I-3-2-2	11.58	4.72
F-3	I-1	13.28	3.17	T-1	I-1	5.90	2.76
	I-1b	11.97	3.65		I-1b	5.71	3.54
	I-2-1	11.04	3.13		I-2-1	7.42	2.81
	I-2-1b	7.96	3.62		I-2-1b	10.41	3.73
	I-2-2	12.26	3.08		I-2-2	9.27	3.09
	I-3-1-2	5.88	3.97		I-3-1-2	12.86	4.16
	I-3-2-2	10.07	3.73		I-3-2-2	13.66	3.76

a The O_vac_ and N notation indicates the sites in the nanoparticle where the oxygen vacancy and the N doping locate, respectively. The *d*_N-Vo_ (in Å) corresponds to the distance between O_vac_ and the N atom.

To get further insight into the nonmetal doping of TiO_2_ NPs, we also performed an analogous analysis for the O_vac_ formation on the *C*_O_ and *C*_i_ doped (TiO_2_)_84_ NP. For this purpose, we have selected the E-2, F-2, and I-3-1 sites because these are the most stable sites for *C*_O_ doping. In addition, we also consider the I-3-2 site to directly compare with previous surface and bulk models.^17,24,26^ Concerning *C*_i_ doping, we considered the I-1, I-2-2, I-3-1-2, and I-3-2-3 positions, as these are the most stable ones. For the *C*_O_-doped NPs, we found the O_vac_ formation energy values in the 2.30–5.12 eV range (Table S2). All cases where the dopant species or oxygen vacancy is located at the I-3-2 site show an O_vac_ formation energy below 3.85 eV. This indicates that sites located in the subsurface as I-3-2 play a key role in the formation of reduced titania nanoparticles, in agreement with previous results for the undoped (TiO_2_)_84_ NP.^38^ On the other hand, for *C*_i_ doping there is almost no effect in the oxygen vacancy energy formation, with values between 3.7 and 5.0 eV. Despite this unfavorable situation, the O_vac_ in the I-3-2 site emerges again as the most favorable thermodynamically, in consistency with the cases of *C*_O_ doping and undoped (TiO_2_)_84_ NPs.

Once the thermodynamic aspects have been discussed, it is necessary to describe the effect of O_vac_ on the electronic structure of the N-doped (TiO_2_)_84_ NP. To make this discussion easy to follow, we focus on the cases where reducibility is facilitated in terms of lowest formation energies. At the same time, we select representative positions from the apical to the equatorial region for substituting oxygen models. For completeness, we comment on both N and C doping. We selected positions labeled as E-2, F-2, F-3, I-3-1, and I-3-2 for *N*_s_, and E-2, F-2, I-3-1, and I-3-2 for *C*_O_, and at least one of each channel at the interstitial models for *N*_i_ and *C*_i_ with positions I-1, I-2-2, I-3-1-2, and I-3-2-2, and I-1, I-2-2, I-3-1-2, and I-3-2-3, for *N*_i_ and *C*_i_, respectively (see Tables S3–S6). In a previous study on the stoichiometric (TiO_2_)_84_ NP, it was reported that the presence of O_vac_ reduced dramatically the energy gap at ∼0.5 eV, as reproduced in Figure S3.^38^ The resulting energy gap for the reduced doped (TiO_2_)_84_ NP is sensitive to the O_vac_ and dopant positions. When O_vac_ locates at the edges (E-2, Figure S4), the resulting energy gap of the N-doped titania nanostructure decreases by 2 eV with respect to that of undoped (TiO_2_)_84_ NP. This reduction is systematically observed regardless of the N position. This situation is even more pronounced in the case of C-doped (TiO_2_)_84_ NP, where the energy gap is reduced by 3 eV with respect to the undoped NP. Thus, C doping has a larger impact on the electronic structure of reduced/doped (TiO_2_)_84_ NP. A similar situation is observed when O_vac_ locates at the face (F-2, Figure S5). Here, C-doped nanostructures expose similar energy gaps (see Figure S3 for comparison). However, for the N-doped nanostructures, the energy gap decrease is larger than in the former case (Figure S5). More interesting is the case where O_vac_ locates in another facet region, labeled as F-3 (Figure S6). The energy gap decrease is analogous regardless of whether the dopant is a C or N atom and of the position they occupy. This result is similar to that where the O_vac_ is in the subsurface region (I-3-2, Figure S8). The energy gap of doped titania nanostructures shows a higher variation when creating an O_vac_ in the presence of the C atom; meanwhile, such variation is less pronounced when the titania nanostructure is N-doped.

## Conclusions

The formation of N-doped titania NPs and the impact of such doping on their reducibility through a neutral oxygen vacancy formation have been computationally studied thoroughly using the bipyramidal (TiO_2_)_84_ NP as a realistic, representative model. For these NPs, N-doping takes place by either substituting one O atom by one N atom, or adding a N atom to the interstitial region. The former requires energies in the 4.70–5.44 eV range, with doping in the inside region being the most favored. However, interstitial doping is even more favorable as it involves formation energies in the 2.35–5.36 eV range. Interstitial N-doping is also preferred in anatase bulk but at a higher cost of 4.3 eV, and this is also the case for the anatase most stable surface, where the predicted cost is of 3.7 eV. From the electronic viewpoint, substitutional N largely stabilizes the LUMO orbital energy with a concomitant reduction of the energy gap of about 2.0 eV. Meanwhile, the most stable interstitial N doping leads to a somewhat small reduction of the energy gap going from 0.41 to 1.88 eV.

Concerning the NPs’ reducibility, substitutional N leads to an oxygen vacancy formation energy in the 1.81–5.15 eV range, and in the 2.11–4.91 eV range for interstitial N. These values are sensibly larger than the 0.6 eV reported for N-doped anatase bulk. A result questioned the efficiency of N-doped titania in photocatalysis because oxygen vacancies act as efficient electron traps. Now, the fact that N doping of anatase titania nanostructures is more favorable than for anatase bulk together with the more difficult reducibility of the N-doped structure, and an additional decrease of the energy gap in the reduced N-doped (TiO_2_)_84_, offers a sound explanation for the observed photocatalytic efficiency of N-doped titania while showing the need for realistic models of these systems.

## Supporting Information Available

Listed the energy gap of (N, C)-doped (TiO_2_)_84_ NP using PBE and PBEx functionals (Table S1); compiles the formation energy of O_vac_ of *C*_O_-doped and *C*_i_-doped (TiO_2_)_84_ NP (Table S2); energy gap of all reduced and *N*_s_-doped (TiO_2_)_84_ NPs investigated including PBE and PBEx results (Table S3); compiles the energy gap of all reduced and *N*_i_-doped (TiO_2_)_84_ NPs investigated including PBE and PBEx results (Table S4); energy gap of reduced and *C*_O_-doped (TiO_2_)_84_ and *C*_i_-doped (TiO_2_)_84_ NPs respectively, using PBE and PBEx functionals (Tables S5 and S6); Kohn–Sham orbital energy level diagram of doped (TiO_2_)_84_ NPs including interstitial and substitution N or C as doped element (Figures S1–S9) (PDF)
