# Supplementary material for: Role of N Doping in the Reduction of Titania Nanostructures
Source: J Phys Chem C Nanomater Interfaces. 2023 Oct 2;127(40):20128–36. doi: 10.1021/acs.jpcc.3c04665 (PMC10578348; doi:10.1021/acs.jpcc.3c04665)
Supplement: Supplementary file 1 — jp3c04665_si_001.pdf [file jp3c04665_si_001.pdf]

Role of N Doping on the Reduction of Titania Nanostructures. †

Elena R. Remesal, Ángel Morales-García,* Francesc Illas

Departament de Ciència de Materials i Química Física & Institut de Química Teòrica i Computacional (IQTCUB), Universitat de Barcelona, c/ Martí i Franquès 1-11, 08028 Barcelona, Spain

e-mail: angel.morales@ub.edu

Table S1. Energy gap and Kohn-Sham orbital energy levels of stoichiometric and doped $(TiO_2)_{84}$ NPs as predicted from DFT based calculations with the PBE and PBEx functional. The last row corresponds to the stoichiometric $(TiO_2)_{84}$ NPs.

		PBE			PBEx		
**Dopant**	**Doping site**	E_g	**LUMO**	**HOMO**	E_g	**LUMO**	**HOMO**
N_s	E-2	1.02	-5.76	-6.78	1.78	-5.27	-7.05
	F-2	0.67	-6.25	-6.92	2.00	-5.62	-7.62
	F-3	0.79	-6.10	-6.89	1.98	-5.65	-7.63
	I-3-1	0.78	-6.61	-7.39	1.88	-6.22	-8.10
	I-3-2	0.71	-6.39	-7.10	1.83	-6.03	-7.87
	T-1	0.77	-5.34	-6.12	2.14	-4.69	-6.83
N_i	I-1	1.24	-5.53	-6.76	2.63	-5.01	-7.64
	I-1b	0.92	-5.46	-6.38	2.04	-4.96	-7.00
	I-2-1	1.14	-6.01	-7.15	2.46	-5.54	-8.00
	I-2-1b	1.13	-5.06	-6.19	2.39	-4.65	-7.04
	I-2-2	0.92	-5.43	-6.35	2.05	-4.95	-7.00
	I-3-1-1	0.07	-4.87	-4.93	0.41	-4.57	-4.98
	I-3-1-2	0.65	-5.18	-5.83	1.68	-4.77	-6.44
	I-3-1-3	1.09	-5.52	-6.60	2.42	-5.08	-7.50
	I-3-2-1	0.97	-5.23	-6.20	2.24	-4.81	-7.05
	I-3-2-2	1.01	-4.98	-5.98	2.14	-4.65	-6.79
	I-3-2-3	1.18	-5.18	-6.37	2.54	-4.73	-7.27
C_o	E-2	0.47	-4.89	-5.36	1.14	-4.62	-5.76
	F-2	0.27	-4.91	-5.18	0.88	-4.63	-5.51
	F-3	0.74	-4.90	-5.65	1.53	-4.62	-6.15
	I-3-1	0.94	-4.95	-5.89	1.70	-4.67	-6.37
	I-3-2	0.67	-5.58	-6.25	1.13	-5.78	-6.92
	T-1	0.68	-4.91	-5.59	0.83	-4.64	-5.47
C_i	I-1	1.42	-4.95	-6.37	2.29	-4.66	-6.96
	I-2-1	1.38	-4.90	-6.29	2.33	-4.61	-6.94
	I-2-2	0.49	-4.94	-5.42	1.19	-4.65	-5.84
	I-3-1-1	0.04	-4.82	-4.86	0.29	-4.58	-4.87
	I-3-1-2	0.11	-4.97	-5.08	0.22	-4.58	-4.79
	I-3-1-3	0.05	-4.81	-4.85	0.31	-4.54	-4.86
	I-3-2-1	1.33	-4.91	-6.24	2.27	-4.62	-6.89
	I-3-2-2	0.08	-4.81	-4.89	0.30	-4.56	-4.87
	I-3-2-3	0.25	-4.91	-5.16	0.92	-4.63	-5.55
**Stoichiometric**	-	2.52	-4.91	-7.43	3.60	-4.62	-8.22

Table S2. Formation energy of the oxygen vacancy (E_{f,v_O}, in eV) of C_O-doped and C_i-doped $(TiO_2)_{84}$ NP. The O_{vac} and N notation indicates the sites in the nanoparticle where the oxygen vacancy and the N doping locates, respectively. The d_{C-V_O} (in Å) corresponds to the distance between O_{vac} and N atom.

O_{vac}	C_o site	d_{C-Vo}	E_{f,v_O}	C_i site	d_{C-Vo}	E_{f,v_O}
E-2	F-2	11.63	4.66	I-1	11.45	4.74
	I-3-1	13.18	4.80	I-2-2	8.08	4.69
	I-3-2	8.0	2.30	I-3-1-2	7.65	4.93
				I-3-2-3	5.73	4.06
F-2	E-2	11.66	4.56	I-1	6.61	4.60
	I-3-1	6.89	5.12	I-2-2	8.11	4.60
	I-3-2	6.9	2.76	I-3-1-2	6.05	4.99
				I-3-2-3	11.14	4.70
F-3	E-2	8.95	4.75	I-1	13.84	4.32
	F-2	9.17	4.49	I-2-2	11.79	4.47
	I-3-1	15.08	4.40	I-3-1-2	5.79	4.90
	I-3-2	7.74	2.47	I-3-2-3	8.89	4.44
I-3-1	E-2	13.37	4.76	I-1	2.67	4.60
	F-2	7.89	4.68	I-2-2	6.33	4.74
	I-3-2	9.66	4.74	I-3-1-2	10.27	4.82
				I-3-2-3	12.68	4.78
I-3-2	E-2	8.11	3.75	I-1	7.46	3.82
	F-2	6.16	3.85	I-2-2	5.06	4.99
	I-3-1	9.44	3.66	I-3-1-2	1.41	3.70
				I-3-2-3	5.10	3.88
T-1	E-2	15.07	4.88	I-1	5.56	4.51
	F-2	9.69	4.69	I-2-2	8.98	4.83
	I-3-1	3.09	4.00	I-3-1-2	12.67	4.90
	I-3-2	12.4	3.77	I-3-2-3	15.21	4.87

Table S3. Energy gap and Kohn-Sham orbital energy level of reduced and N_s-doped $(TiO_2)_{84}$ NPs as predicted from DFT based calculations with the PBE and PBEx functional. For the PBEx simulation, representative positions are selected from the apical to the equatorial region.

		PBE			PBEx		
**O_{vac}**	**N_s position**	**E_g**	**LUMO**	**HOMO**	**E_g**	**LUMO**	**HOMO**
**E-2**	F-2	0.44	-4.95	-5.39	1.55	-4.67	-6.22
	F-3	0.44	-4.87	-5.31			
	I-3-1	0.42	-5.02	-5.44	1.52	-4.74	-6.26
	I-3-2	0.43	-4.89	-5.32	1.54	-4.60	-6.13
**F-2**	E-2	0.30	-4.90	-5.20			
	F-3	0.30	-4.87	-5.16			
	I-3-1	0.22	-5.01	-5.23	1.04	-4.73	-5.77
	I-3-2	0.23	-4.88	-5.10	1.06	-4.58	-5.64
**F-3**	E-2	0.08	-4.90	-4.98			
	F-2	0.08	-4.94	-5.02	0.44	-4.67	-5.12
	I-3-1	0.08	-5.02	-5.10	0.44	-4.76	-5.20
	I-3-2	0.09	-4.88	-4.97	0.47	-4.60	-5.07
**I-3-1**	E-2	0.66	-5.74	-6.40			
	F-2	1.13	-5.59	-6.71	2.18	-5.19	-7.37
	F-3	1.13	-5.75	-6.88			
	I-3-2	1.13	-5.70	-6.83	2.18	-5.30	-7.48
	T-1	0.40	-5.22	-5.6226			
**I-3-2**	E-2	0.08	-4.86	-4.94	0.47	-4.54	-5.01
	F-2	0.08	-4.90	-4.97	0.47	-4.58	-5.05
	F-3	0.03	-4.85	-4.89	0.35	-4.57	-4.92
	I-3-1	0.08	-4.97	-5.05	0.47	-4.66	-5.13
**T-1**	E-2	0.94	-5.26	-6.21			
	F-2	0.94	-5.10	-6.04	2.57	-4.47	-7.03
	F-3	0.94	-5.27	-6.21			
	I-3-1	0.26	-4.81	-5.07	1.47	-4.52	-5.99
	I-3-2	0.94	-5.21	-6.15	2.57	-4.59	-7.15

Table S4. Energy gap and Kohn-Sham orbital energy level of reduced and N_i-doped $(TiO_2)_{84}$ NPs as predicted from DFT based calculations with the PBE and PBEx functional. For the PBEx simulation the selection of at least one representative positions of each channel is achieved.

		PBE			PBEx		
**O_{vac}**	**N_i position**	**E_g**	**LUMO**	**HOMO**	**E_g**	**LUMO**	**HOMO**
**E-2**	I-1	0.42	-5.00	-5.42	1.52	-4.72	-6.24
	I-1b	0.42	-4.99	-5.41			
	I-2-1	1.44	-5.03	-6.48			
	I-2-1b	0.41	-4.91	-5.33			
	I-2-2	0.41	-4.95	-5.36	1.51	-4.67	-6.17
	I-3-1-2	0.36	-4.90	-5.26	1.43	-4.53	-5.96
	I-3-2-2	0.36	-4.90	-5.26	1.43	-4.62	-6.04
**F-2**	I-1	0.24	-4.98	-5.22	1.06	-4.70	-5.76
	I-1b	0.24	-4.96	-5.20			
	I-2-1	0.19	-4.98	-5.18			
	I-2-1b	0.27	-4.90	-5.18			
	I-2-2	0.25	-4.94	-5.18	1.07	-4.65	-5.72
	I-3-1-2	0.07	-4.87	-4.94	0.41	-4.62	-5.03
	I-3-2-2	0.33	-4.87	-5.20	1.07	-4.63	-5.70
**F-3**	I-1	0.08	-5.00	-5.08	0.26	-4.59	-4.86
	I-1b	0.10	-4.97	-5.07			
	I-2-1	0.08	-5.01	-5.09			
	I-2-1b	0.09	-4.91	-5.00			
	I-2-2	0.09	-4.94	-5.03	0.46	-4.68	-5.14
	I-3-1-2	0.10	-4.84	-4.94	0.50	-4.54	-5.04
	I-3-2-2	0.07	-4.89	-4.96	0.42	-4.64	-5.06
**I-3-1**	I-1	1.10	-5.14	-6.24	2.17	-4.71	-6.88
	I-1b	0.85	-5.20	-6.05			
	I-2-1	0.97	-5.06	-6.03			
	I-2-1b	0.48	-5.09	-5.57			
	I-2-2	0.17	-5.41	-5.58	1.22	-4.92	-6.15
	I-3-1-2	0.47	-5.18	-5.65	1.46	-4.76	-6.22
	I-3-2-2	0.70	-4.95	-5.65	1.58	-4.64	-6.22
**I-3-2**	I-1	0.08	-4.95	-5.03	0.47	-4.63	-5.11
	I-1b	0.08	-4.94	-5.02			
	I-2-1	0.21	-4.85	-5.06	0.46	-4.67	-5.13
	I-2-1b	0.08	-4.87	-4.95			
	I-2-2	0.08	-4.90	-4.97	0.47	-4.58	-5.05
	I-3-1-2	0.85	-4.87	-5.72	0.18	-4.65	-4.83
	I-3-2-2	0.04	-4.88	-4.91	0.40	-4.56	-4.96
**T-1**	I-1	0.80	-4.79	-5.58	2.06	-4.49	-6.55
	I-1b	0.86	-4.82	-5.68			
	I-2-1	0.58	-4.83	-5.41			
	I-2-1b	0.78	-5.19	-5.97			
	I-2-2	0.68	-5.13	-5.81	1.84	-4.51	-6.35

	I-3-1-2	0.09	-5.30	-5.39	1.11	-4.69	-5.80
	I-3-2-2	0.35	-5.29	-5.63	1.52	-4.67	-6.19

Table S5. Energy gap and Kohn-Sham orbital energy level of reduced and C_O-doped $(TiO_2)_{84}$ NPs as predicted from DFT based calculations with the PBE and PBEx functional.

		PBE			PBEx		
**O_{vac}**	**C_o position**	**E_g**	**LUMO**	**HOMO**	**E_g**	**LUMO**	**HOMO**
**E-2**	F-2	0.12	-4.84	-4.97	0.48	-4.60	-5.08
	I-3-1	0.13	-4.88	-5.02	0.50	-4.57	-5.07
	I-3-2	0.62	-5.34	-5.96	1.39	-5.05	-6.43
**F-2**	E-2	0.04	-4.86	-4.90	0.25	-4.65	-4.90
	I-3-1	0.13	-4.94	-5.07	0.77	-4.66	-5.44
	I-3-2	1.15	-4.89	-6.04	1.96	-4.56	-6.52
**F-3**	E-2	0.06	-4.82	-4.88	0.40	-4.53	-4.93
	F-2	0.06	-4.81	-4.87	0.42	-4.51	-4.93
	I-3-1	0.00	-4.94	-4.94	0.41	-4.59	-4.99
	I-3-2	1.08	-5.00	-6.08	1.83	-4.71	-6.55
**I-3-1**	E-2	0.46	-4.88	-5.34	1.13	-4.61	-5.74
	F-2	0.28	-4.91	-5.18	0.90	-4.63	-5.53
	I-3-2	0.06	-5.57	-5.63	0.95	-5.27	-6.22
**I-3-2**	E-2	0.02	-4.83	-4.85	0.20	-4.58	-4.78
	F-2	0.07	-4.77	-4.84	0.27	-4.56	-4.83
	I-3-1	*			0.24	-4.59	-4.83
**T-1**	E-2	*			0.13	-4.54	-4.68
	F-2	0.00	-4.73	-4.73	0.11	-4.57	-4.69
	I-3-1	*			0.16	-4.49	-4.65
	I-3-2	0.21	-5.65	-5.85	0.26	-5.84	-6.09

*Absence of energy gap

Table S6. Energy gap and Kohn-Sham orbital energy level of reduced and C_i-doped $(TiO_2)_{84}$ NPs as predicted from DFT based calculations with the PBE and PBEx functional.

		PBE			PBEx		
**O_{vac}**	**C_i position**	**E_g**	**LUMO**	**HOMO**	**E_g**	**LUMO**	**HOMO**
**E-2**	I-1	0.13	-4.87	-5.00	0.45	-4.57	-5.02
	I-2-2	0.12	-4.86	-4.97	0.44	-4.62	-5.05
	I-3-2-3	0.07	-4.79	-4.86	0.48	-4.49	-4.97
**F-2**	I-1	0.03	-4.95	-4.98	0.27	-4.70	-4.96
	I-2-2	0.04	-4.87	-4.91	0.29	-4.62	-4.90
	I-3-1-2	0.05	-4.78	-4.83	0.27	-4.62	-4.90
	I-3-2-3	0.04	-4.86	-4.91	0.21	-4.68	-4.90
**F-3**	I-1	0.07	-4.87	-4.94	0.41	-4.57	-4.98
	I-2-2	0.05	-4.85	-4.90	0.42	-4.54	-4.96
	I-3-1-2	0.03	-4.74	-4.77	0.32	-4.49	-4.81
	I-3-2-3	0.04	-4.82	-4.86	0.41	-4.52	-4.93
**I-3-1**	I-1	0.50	-4.92	-5.42	1.32	-4.62	-5.94
	I-2-2	0.51	-4.94	-5.45	1.21	-4.66	-5.86
	I-3-1-2	0.04	-4.79	-4.83	0.21	-4.61	-4.83
	I-3-2-3	0.24	-4.92	-5.16	0.91	-4.63	-5.54
**I-3-2**	I-1	0.02	-4.90	-4.91	0.18	-4.67	-4.85
	I-2-2	0.10	-4.94	-5.04	0.82	-4.65	-5.47
	I-3-1-2	0.01	-4.83	-4.84	0.23	-4.56	-4.79
	I-3-2-3	0.02	-4.83	-4.85	0.22	-4.59	-4.80
**T-1**	I-1	0.20	-4.82	-5.02	0.87	-4.54	-5.41
	I-2-2	*			0.12	-4.60	-4.71
	I-3-1-2	0.01	-4.70	-4.71	0.17	-4.55	-4.72
	I-3-2-3	*			0.13	-4.58	-4.71

* Absence of energy gap

Figure S1. Kohn-Sham orbital energy level diagram of C_O-doped $(TiO_2)_{84}$ NPs obtained from DFT based calculations with the hybrid PBEx functional. The dotted lines correspond to Kohn-Sham orbital energy levels of the stoichiometric $(TiO_2)_{84}$ NP.

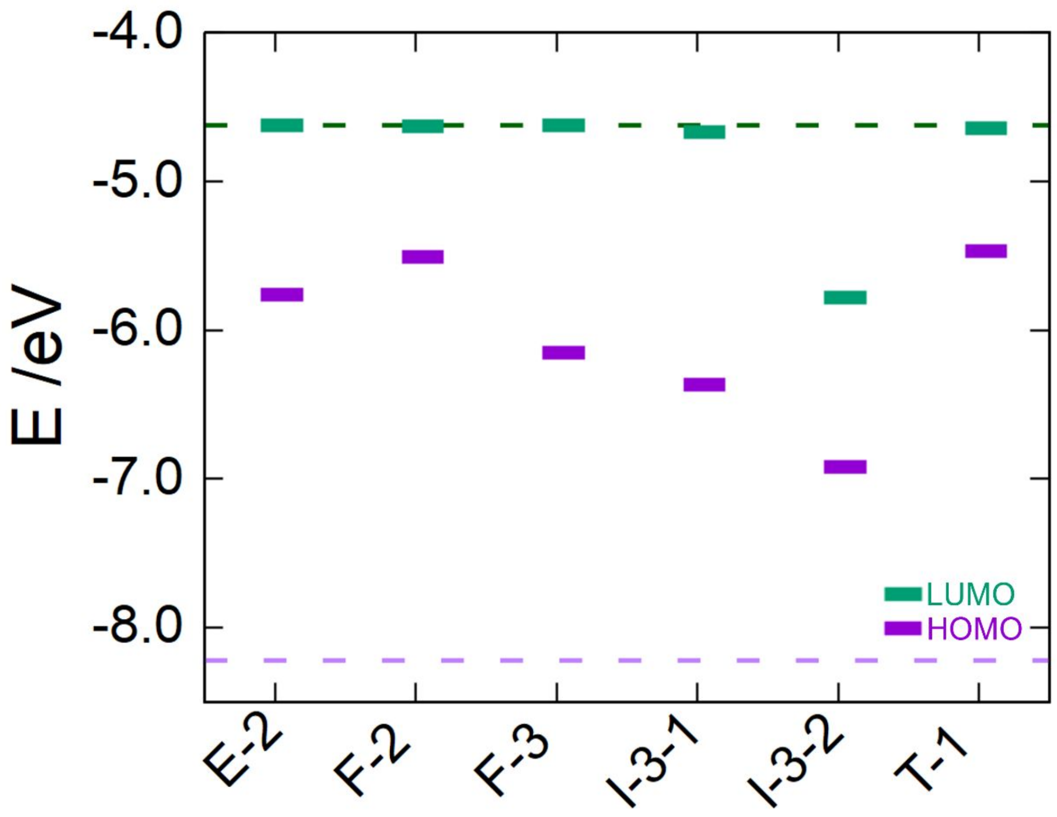

Figure S2. Kohn-Sham orbital energy level diagram of C_i-doped $(TiO_2)_{84}$ NPs obtained from DFT based calculations with the hybrid PBEx functional. The dotted lines correspond to Kohn-Sham orbital energy levels of the stoichiometric $(TiO_2)_{84}$ NP.

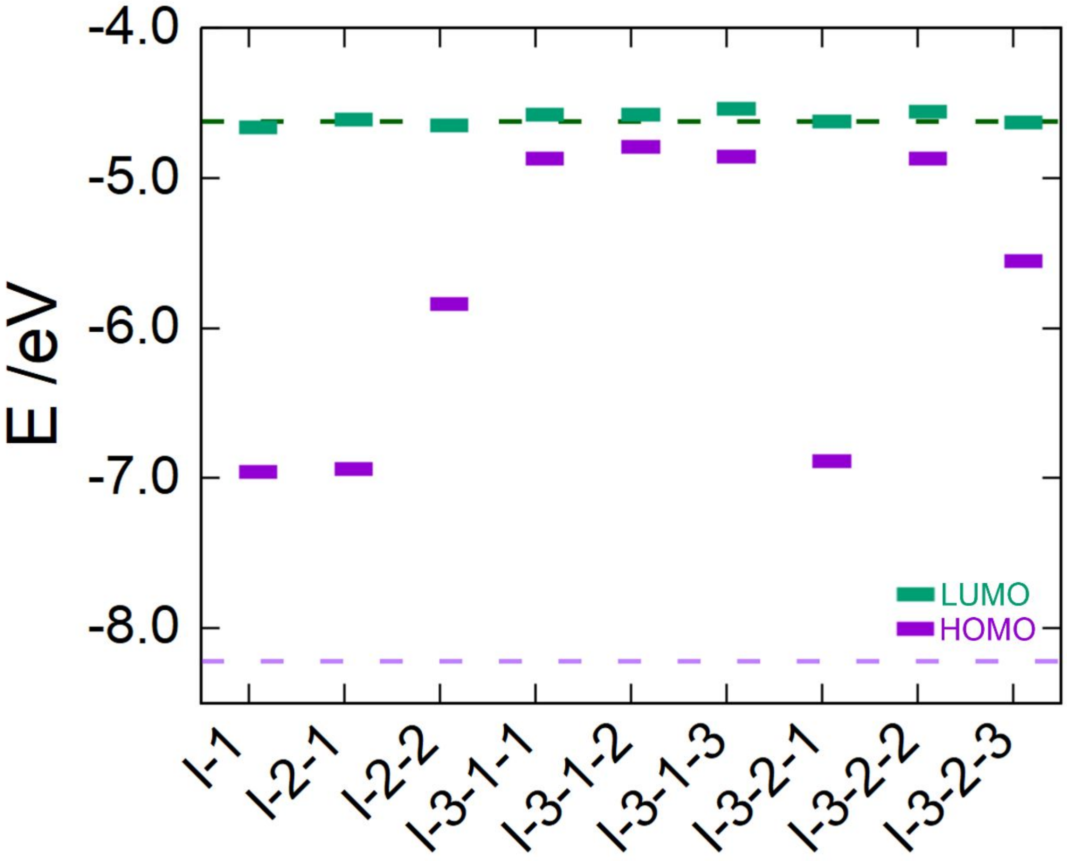

Figure S3. Kohn-Sham orbital energy level diagram of reduced ($Ti_{84}O_{167}$) NP as predicted from DFT based calculations with the PBEx functional. The dotted lines correspond to Kohn-Sham orbital energy levels of the stoichiometric $(TiO_2)_{84}$ NP.[1]

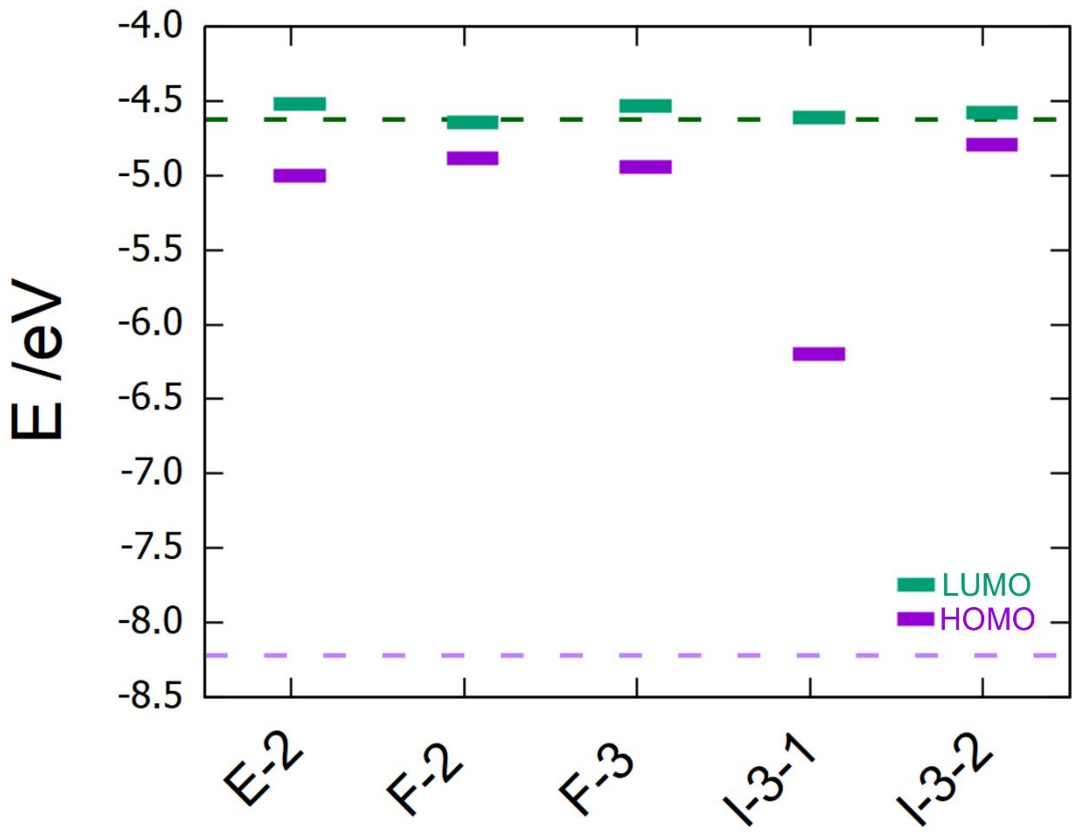

Figure S4. Kohn-Sham orbital energy level diagram of doped and reduced (N_s-$Ti_{84}O_{166}$ and N_i-$Ti_{84}O_{167}$; C_O-$Ti_{84}O_{166}$ and C_i-$Ti_{84}O_{167}$) NPs and oxygen vacancy position labelled E-2, as predicted from DFT based calculations with the PBEx functional. The dotted lines correspond to Kohn-Sham orbital energy levels of the stoichiometric $(TiO_2)_{84}$ NP.

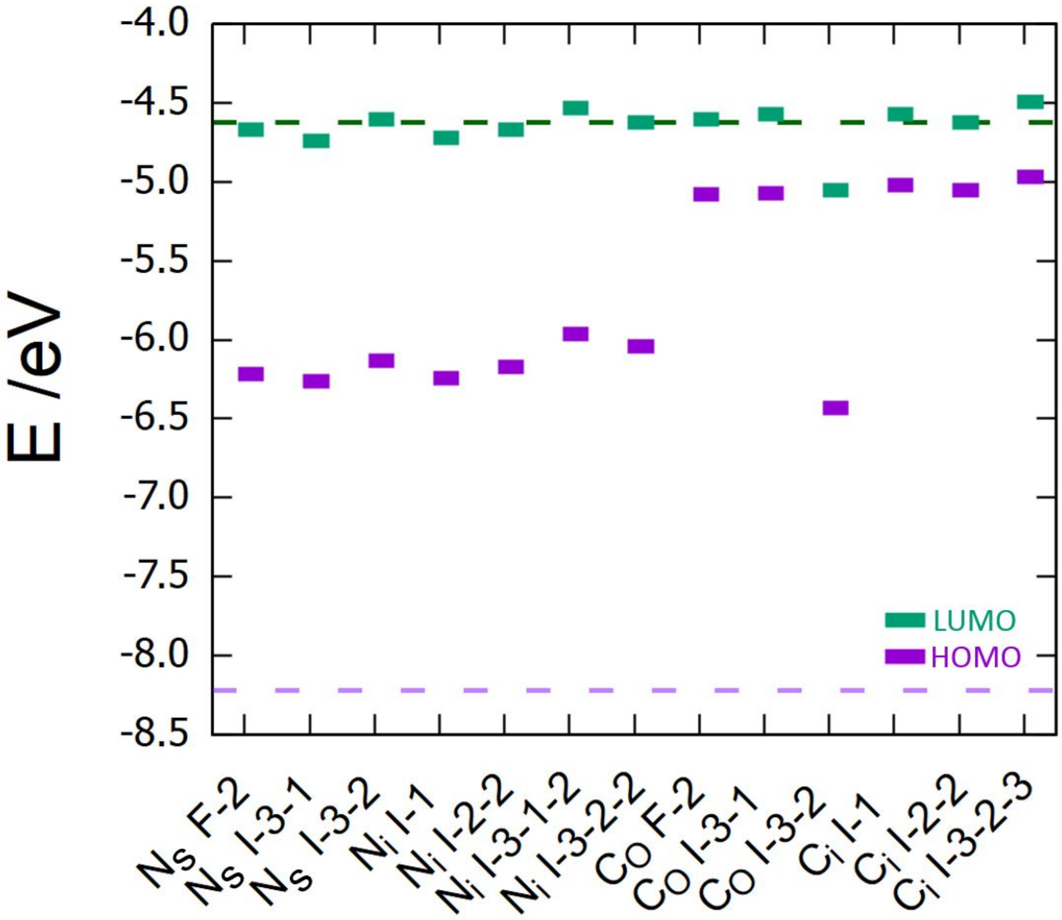

Figure S5. Kohn-Sham orbital energy level diagram of doped and reduced (N_s-$Ti_{84}O_{166}$ and N_i-$Ti_{84}O_{167}$; C_O-$Ti_{84}O_{166}$ and C_i-$Ti_{84}O_{167}$) NPs and oxygen vacancy position labelled F-2, as predicted from DFT based calculations with the PBEx functional. The dotted lines correspond to Kohn-Sham orbital energy levels of the stoichiometric $(TiO_2)_{84}$ NP.

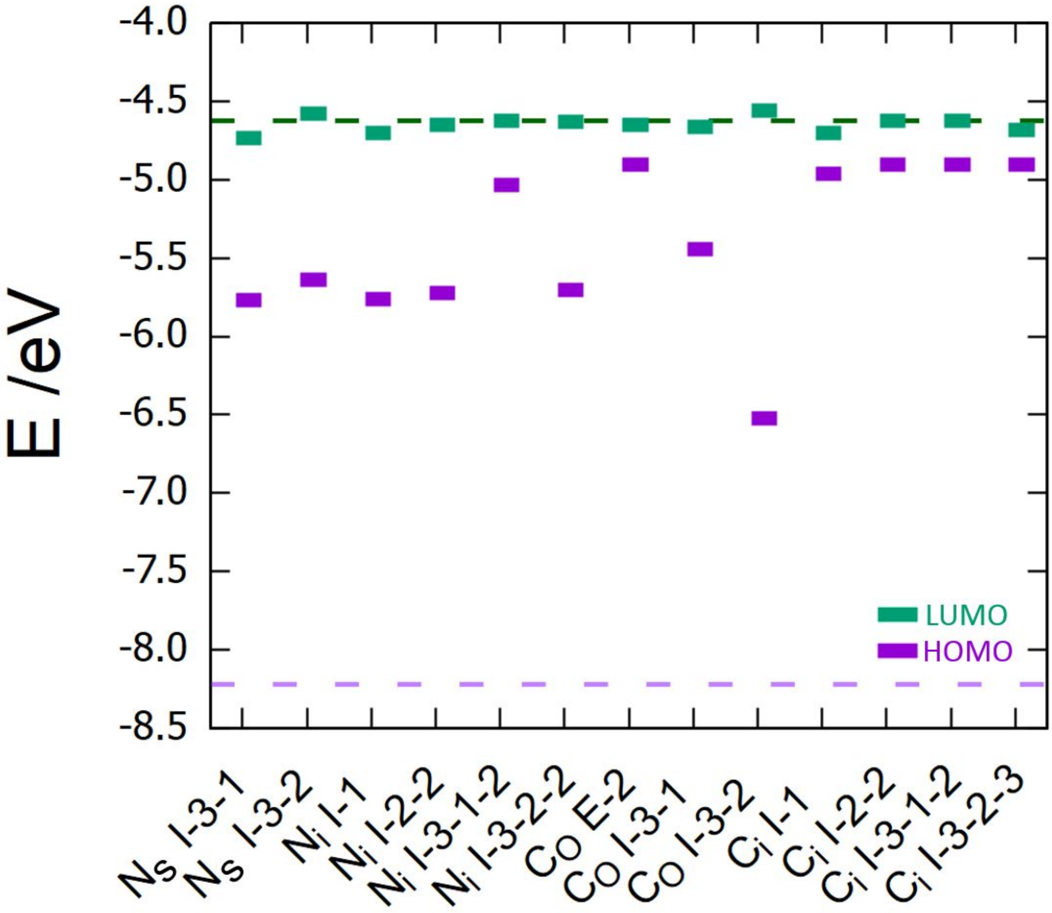

Figure S6. Kohn-Sham orbital energy level diagram of doped and reduced (N_s-$Ti_{84}O_{166}$ and N_i-$Ti_{84}O_{167}$; C_O-$Ti_{84}O_{166}$ and C_i-$Ti_{84}O_{167}$) NPs and oxygen vacancy position labelled F-3, as predicted from DFT based calculations with the PBEx functional. The dotted lines correspond to Kohn-Sham orbital energy levels of the stoichiometric $(TiO_2)_{84}$ NP.

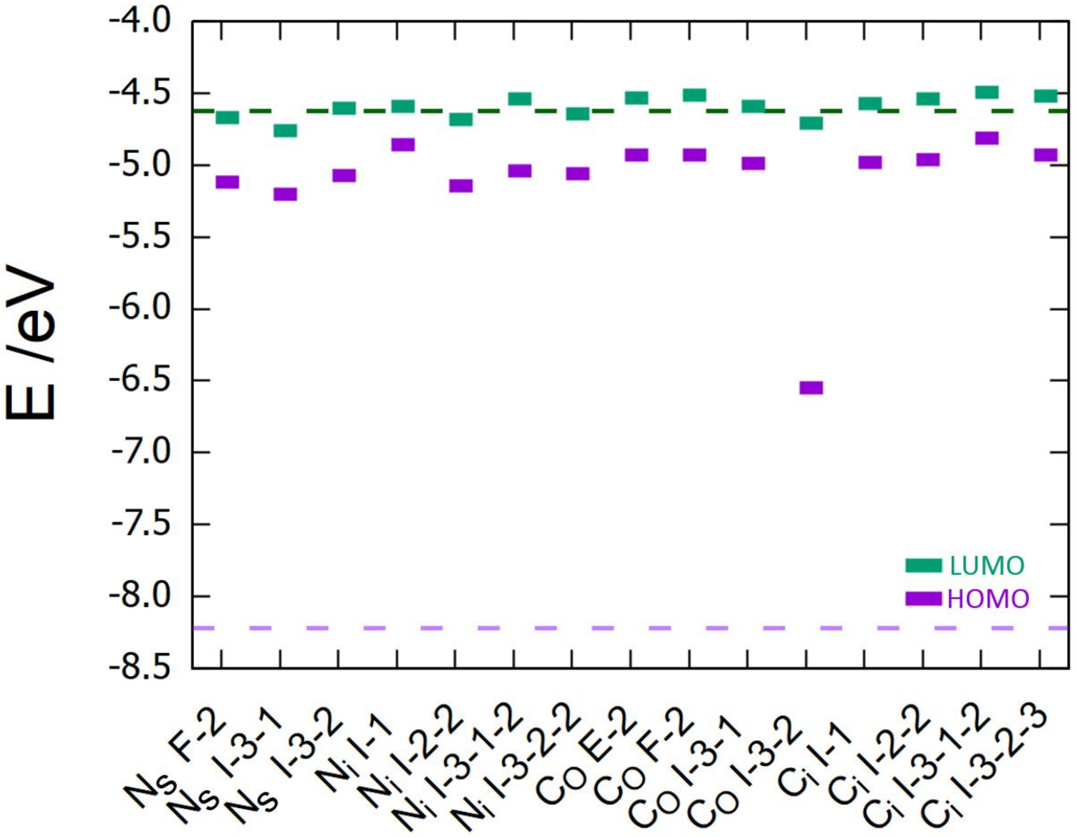

Figure S7. Kohn-Sham orbital energy level diagram of doped and reduced (N_s-$Ti_{84}O_{166}$ and N_i-$Ti_{84}O_{167}$; C_O-$Ti_{84}O_{166}$ and C_i-$Ti_{84}O_{167}$) NPs and oxygen vacancy position labelled as I-3-1, as predicted from DFT based calculations with the PBEx functional. The dotted lines correspond to Kohn-Sham orbital energy levels of the stoichiometric $(TiO_2)_{84}$ NP.

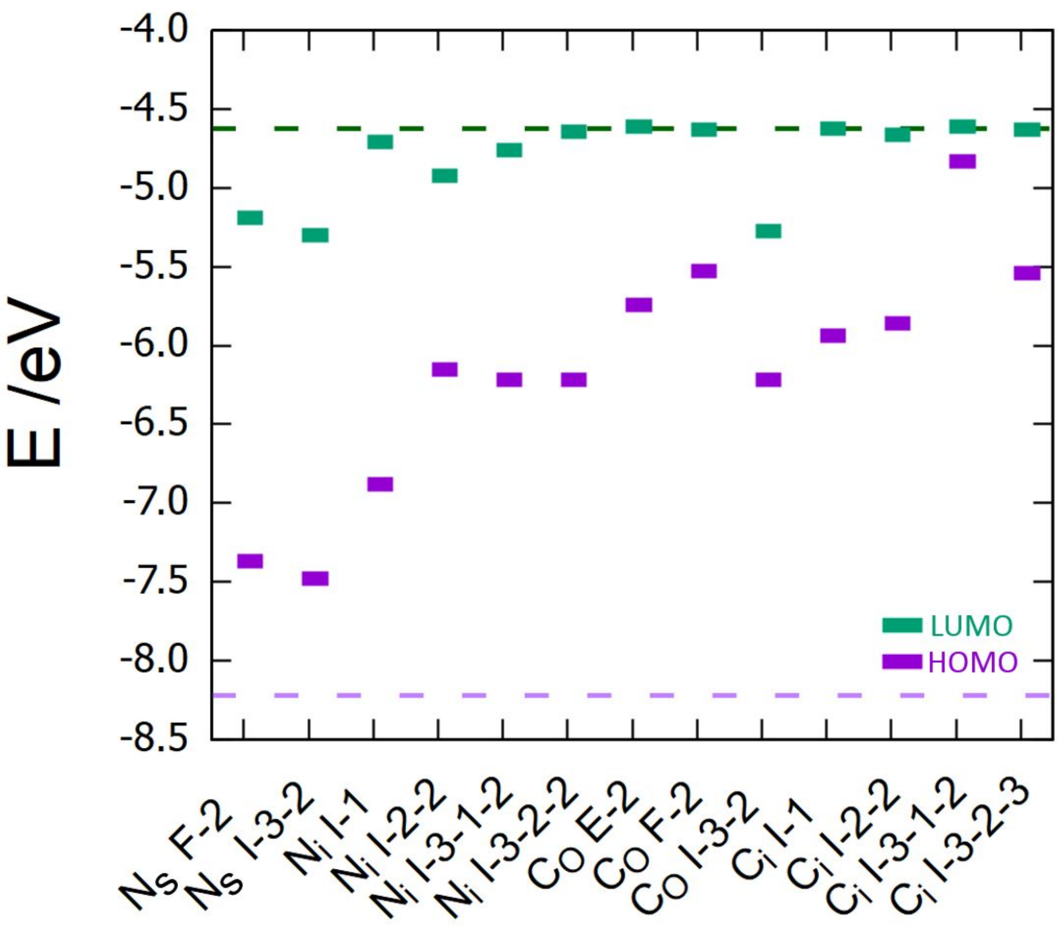

Figure S8 Kohn-Sham orbital energy level diagram of doped and reduced (N_s-$Ti_{84}O_{166}$ and N_i-$Ti_{84}O_{167}$; C_O-$Ti_{84}O_{166}$ and C_i-$Ti_{84}O_{167}$) NPs and oxygen vacancy position labelled I-3-2, as predicted from DFT based calculations with the PBEx functional. The dotted lines correspond to Kohn-Sham orbital energy levels of the stoichiometric $(TiO_2)_{84}$ NP.

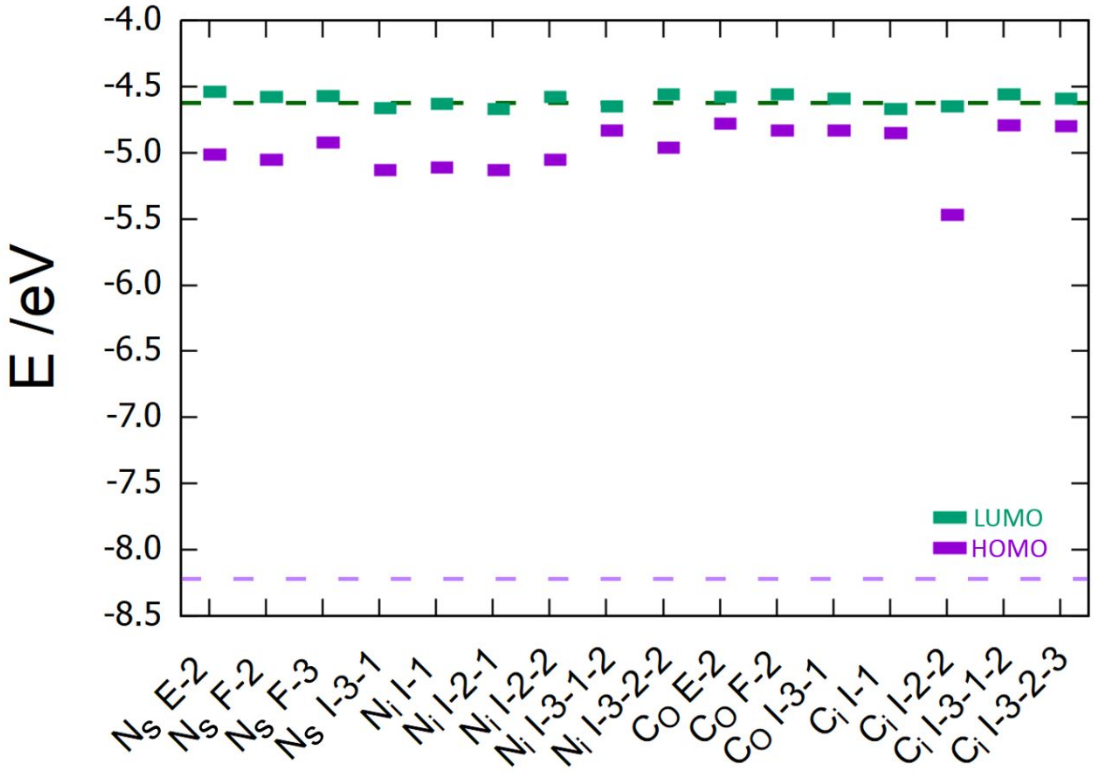

Figure S9. Kohn-Sham orbital energy level diagram of doped and reduced (N_s-$Ti_{84}O_{166}$ and N_i-$Ti_{84}O_{167}$; C_O-$Ti_{84}O_{166}$ and C_i-$Ti_{84}O_{167}$) NPs and oxygen vacancy position labelled T-1, as predicted from DFT based calculations with the PBEx functional. The dotted lines correspond to Kohn-Sham orbital energy levels of the stoichiometric $(TiO_2)_{84}$ NP.

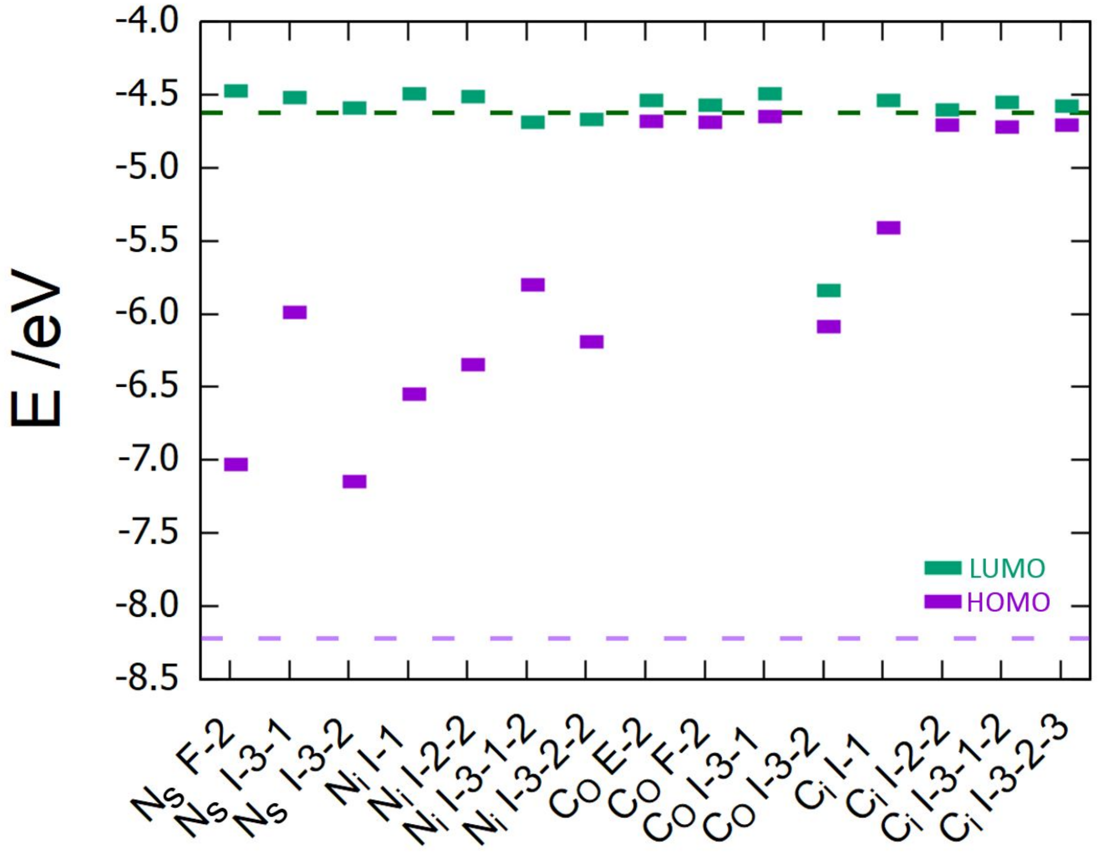

---

[1] Morales-García, A.; Lamiel-García, O-; Valero, R.; Illas, F. *J. Phys. Chem. C* **2018**, *122*, 2413−2421.
